# Multimodal Electrocorticogram Active Electrode Array Based on Zinc Oxide‐Thin Film Transistors

**DOI:** 10.1002/advs.202204467

**Published:** 2022-11-20

**Authors:** Fan Zhang, Luxi Zhang, Jie Xia, Wanpeng Zhao, Shurong Dong, Zhi Ye, Gang Pan, Jikui Luo, Shaomin Zhang

**Affiliations:** ^1^ Key Laboratory of Biomedical Engineering of Ministry of Education Qiushi Academy for Advanced Studies Zhejiang Provincial Key Laboratory of Cardio‐Cerebral Vascular Detection Technology and Medicinal Effectiveness Appraisal Zhejiang University 38 Zheda Road Hangzhou 310027 China; ^2^ College of Information Science and Electronic Engineering Frontier Center of Brain Science and Brain‐machine Integration Cancer Center Zhejiang University 38 Zheda Road Hangzhou 310027 China; ^3^ College of Information Science and Electronic Engineering Zhejiang University 38 Zheda Road Hangzhou 310027 China; ^4^ College of Computer Science and Technology Zhejiang University 38 Zheda Road Hangzhou 310027 China

**Keywords:** transparent electrodes, thin‐film‐transistors, electrocorticogram, neural recording, optogenetic

## Abstract

Active electrocorticogram (ECoG) electrodes can amplify weak electrophysiological signals and improve anti‐interference ability; however, traditional active electrodes are opaque and cannot realize photoelectric collaborative observation. In this study, an active and fully transparent ECoG array based on zinc oxide thin‐film transistors (ZnO TFTs) is developed as a local neural signal amplifier for electrophysiological monitoring. The transparency of the proposed ECoG array is up to 85%, which is superior to that of the previously reported active electrode arrays. Various electrical characterizations have demonstrated its ability to record electrophysiological signals with a higher signal‐to‐noise ratio of 19.9 dB compared to the Au grid (13.2 dB). The high transparency of the ZnO‐TFT electrode array allows the concurrent collection of high‐quality electrophysiological signals (32.2 dB) under direct optical stimulation of the optogenetic mice brain. The ECoG array can also work under 7‐Tesla magnetic resonance imaging to record local brain signals without affecting brain tissue imaging. As the most transparent active ECoG array to date, it provides a powerful multimodal tool for brain observation, including recording brain activity under synchronized optical modulation and 7‐Tesla magnetic resonance imaging.

## Graphical abstract

An electrocorticogram active transparent electrode array that uses zinc oxide thin‐film transistors as a local neural signal amplifier is proposed. As the most transparent active ECoG array to date, it allows monitoring of electrophysiological signals with synchronized optical modulation, 7‐Tesla magnetic resonance imaging, and optical imaging, providing a powerful multimodal tool for brain research.

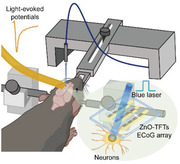

## 1 Introduction

Electrocorticography (ECoG) collects electrophysiological signals from the cerebral cortex through a planar electrode array above or below the dura mater.^[^ ^1^ ^]^ ECoG signals have been used to monitor and locate neurological dysfunctions such as intractable epilepsy.^[^ ^2^ ^]^ In the past few years, as an emerging technology, brain‐computer interface combined with ECoG has helped develop closed‐loop systems that can be used in neuroprosthetic applications for motor functional reconstruction,^[^ ^3^ ^]^ and decoding of vision,^[^ ^4^ ^]^ movements,^[^ ^5^ ^]^ and speech.^[^ ^6^ ^]^ Various micro electrode arrays have been developed to enable highly reliable neural mapping in the cerebral cortex with high spatiotemporal resolution, without damaging the brain tissues.^[^ ^7^, ^8^ ^]^

For wired ECoG electrodes, neural signals are transmitted through wires before being amplified and processed by external circuits, significantly sensitizing them to environmental noise.^[^ ^9^ ^]^ Thus, active electrode arrays^[^ ^10^ ^]^ have been studied and developed to improve the signal‐to‐noise ratio (SNR) using on‐chip preamplifiers that mainly consist of transistors. In active devices, the neuronal transmembrane current, conducted through the electrolyte, polarizes the gate electrodes and changes the conductivity of the active channels by forming local amplifiers.^[^ ^11^ ^]^ Several types of transistors, such as solution‐gated field effect transistors (SGFETs),^[^ ^12^ ^]^ organic electrochemical transistors (OECTs),^[^ ^11^, ^13^ ^]^ and ion‐gated organic electrochemical transistors,^[^ ^14^ ^]^ have been adopted to monitor neural activity, and demonstrate the advantages of transistor‐based active electrode arrays in terms of low noise level.

With the development of optical neuromodulation and imaging techniques in neuroscience, the demand for optically transparent electrode arrays is rapidly increasing. A transparent electrode array can provide an interface that is feasible for simultaneous in situ optical modulation and electrical monitoring. Therefore, these active electrode arrays, including transparent electrodes and active circuits comprising amplifiers and interconnects, are required to be highly transparent. Although some conductive transparent materials in passive electrode arrays, such as ultra‐thin metal films with nanometer thickness,^[^ ^15^, ^16^ ^]^ like indium‐tin‐oxide (ITO),^[^ ^17^, ^18^ ^]^ graphene,^[^ ^19^, ^20^ ^]^ PEDOT:PSS,^[^ ^21^ ^]^ etc., offer possible solutions to transparent electrode arrays, until now, no highly transparent active electrode array has been used because of the difficulties discussed below.

First, the small spontaneous electrophysiological potential of neurons (tens to hundreds of microvolts) and photoexcited potentials (tens to thousands of microvolts) are easily diminished in light‐induced artifacts caused by photosensitive transparent materials.^[^ ^22^, ^23^ ^]^ Therefore, the electrical and optical properties of the devices need to be stable under optical stimulation of different wavelengths and powers for the neuron signal recordings to be unaffected. Second, since the transparency and conductivity of materials often require a compromise,^[^ ^24^ ^]^ complicated structural design and extensive engineering must be followed to select appropriate transparent conductive materials and combine them with transparent semiconductor materials for device fabrication. Furthermore, the structure and processing techniques should be compatible with those of the ECoG array, and transistors produced by the same process should possess stable electrical and optical performance. Additionally, for in vivo applications, the characteristics required of transistors, such as biocompatibility,^[^ ^25^ ^]^ long‐term stability,^[^ ^26^ ^]^ etc., hinder the invention of an appropriate solution for transparent transistors. In 2017, Lee et al. implemented a transparent active electrode array via OECTs and achieved transparency of 60% through gold wires.^[^ ^13^ ^]^ This is the only study to date on transparent active ECoG grids; however, the transparency of the electrode array is limited by the use of ultrathin metal grids.

The high transparency of thin‐film transistor (TFT) makes TFT‐based transparent active ECoG electrode arrays feasible and very attractive. A TFT, a type of FET, is composed of thin‐film elements on a substrate^[^ ^27^ ^]^ and is usually used in large‐area electronic products, such as liquid crystal displays^[^ ^28^ ^]^ and light‐emitting displays.^[^ ^29^ ^]^ Some transparent conducting oxide materials, such as ITO,^[^ ^30^ ^]^ ZnO,^[^ ^31^, ^32^ ^]^ and cadmium oxide,^[^ ^33^ ^]^ have been incorporated to achieve fully transparent TFTs. ZnO is considered to be the most suitable material for the active channel layer in TFTs because of its excellent semiconductor characteristics (high field effective mobility) and high optical transparency (insensitivity to visible light owing to its wide bandgap).^[^ ^34^ ^]^ This provides a potential solution for highly transparent active electrode arrays for neural electrophysiology.

In this study, a transparent active electrode array for neural signal recording on the brain surface with ZnO TFTs as the preamplifiers (we name it ZnO‐TFT electrode array) was developed, and their characteristics and performances were systematically investigated. ZnO TFTs included an ITO conductor, aluminum oxide (Al_2_O_3_) insulator, and ZnO semiconductor and were passivated by an Al_2_O_3_ layer with a thickness of tens of nanometers. The ZnO TFTs exhibited stable electrical properties and a high optical transparency of up to 85% in the visible light range. The ZnO‐TFT electrode array was then employed to record spontaneous sleep waveforms, which demonstrated its feasibility of neural signal acquisition. Owing to the adoption of preamplifiers, the SNR of our ZnO‐TFT electrode array reached 19.9 dB, which is superior to that of the passive Au electrodes (13.2 dB). Owing to its high transparency, it enabled in situ optical stimulation and simultaneous neural electrophysiological recording to map out the light‐evoked potential with SNR of 32.2 dB in an optogenetic mouse brain. Furthermore, the ECoG array was applied to the rat brain in a 7‐Tesla (7T) ultra‐high magnetic field, and a clear magnetic resonance imaging (MRI) with no blurring was obtained for demonstration of its feasibility and potential in MRI‐ECoG synchronous recording.

## 2 Results and Discussion

### 2.1 Structure of ZnO‐TFT Electrode Array

**Figure 1:**
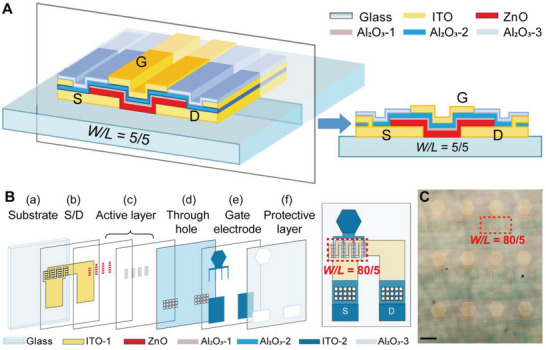
Schematic illustration and layout of the transparent ZnO‐TFT array. A) 3D structure diagram (left) and the cross‐section (right) of the transparent ZnO TFT. B) Left: the exploded view of a ZnO‐TFT electrode, including a) the substrate of 100‐µm thick glass, b) the first ITO layer of 100‐nm thickness for source and drain, c) 20‐nm thick ZnO active layer and the first Al_2_O_3_ layer of 10‐nm thickness for the ZnO layer protection, d) the second Al_2_O_3_ layer of 20‐nm thickness as the gate dielectric layer and through‐holes punched in the layer, e) the second ITO layer of 100‐nm thickness for the gate electrodes, and f) the third Al_2_O_3_ layer of 20‐nm thickness to protect the whole device. Right: the structure of a ZnO‐TFT electrode. The red dashed square labels a transistor with *W/L* = 80/5, which consist of 16 transistors with *W/L* = 5/5 in parallel. C) Microscopic image of a 3 × 4 ZnO‐TFT array. The red dashed frame labels an active region consisting of 16 paralleled ZnO TFT, corresponding to that in the red dashed frame of (B) (right). Scale bar: 200 µm.

To enable a neural interface with high transparency and SNR, we developed active and non‐penetrating electrodes based on transparent ZnO TFTs. The structure of the ZnO TFT is shown in **Figure** **1**A. The ZnO‐TFT electrode array was fabricated using a multi‐layer process, as discussed in Section 4 and Figure S1, Supporting Information.

The cross‐section of the ZnO‐TFT electrode is shown in Figure 1B. The size of ZnO‐TFT array is 10.8 × 7.0 mm^2^, and each array consists of 3 × 4 electrodes with horizonal and vertical spacings of 350 and 620 µm, respectively, as shown in Figure S2, Supporting Information. An image of the ZnO‐TFT electrode array is shown in Figure 1C.

### 2.2 Design and Characterization of ZnO‐TFT Array

**Figure 2:**
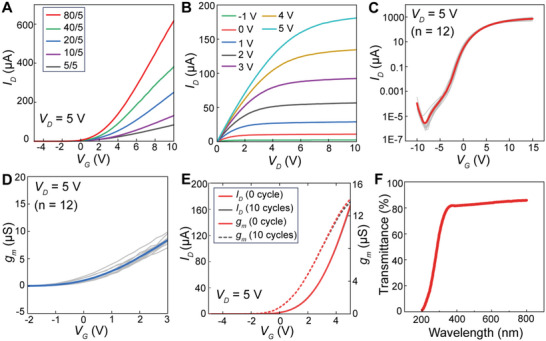
Electrical, mechanical, and optical properties of the ZnO‐TFT array. A) Transfer curves of the TFTs with different *W/L* ratios at *V*_D_ = 5 V. B) Output characteristics of a ZnO‐TFT electrode with *W/L* = 80/5, showing the drain current (*I*_D_) as a function of *V*_D_ with *V*_G_ varying from −1 V to 5 V (step = 1 V). C) Transfer characteristics of 12 ZnO‐TFT electrodes of an array at *V*_D_ = 5 V. The red line is the average transfer curve of the 12 ZnO TFTs. D) The resulting transconductance of 12 ZnO‐TFT electrodes from the same array at *V*_D_ = 5 V. The blue line is the average transfer curve of the 12 ZnO TFTs. E) ZnO‐TFT electrode maintained stable electrical properties after 10 bending cycles at a bending radius of 15 cm. The transfer characteristics were measured at *V*_D_ = 5 V. F) Transmittance spectrum of the ZnO‐TFT array.

Simulations were conducted to optimize the performance of the ZnO TFT as preamplifiers. The width‐to‐length ratios (*W/L*) for the TFTs in the simulation were 5/5, 10/5, 20/5, 40/5, and 80/5 (units: µm). The simulated transfer characteristics and transconductance *g*_m_ are shown in Figures S3A,B, respectively, Supporting Information, which show that after the determination of the quiescent operating point, the transconductance exhibited a linear relationship with *W/L* in the linear region and then saturated with an increase in the *W/L* ratio. We fabricated ZnO TFTs with different *W/L* ratios and characterized their electrical properties (Figure S4A,B, Supporting Information). As expected, the transfer characteristics of the fabricated ZnO TFTs with larger *W/L* ratios exhibited larger linear slopes (**Figure** **2**A), which resulted in a larger *g*_m_. The discrepancies between the experimental and simulation results were mainly attributed to deuterium plasma treatment, by which the threshold voltages of the transistors were reduced to values lesser than those in the simulation, changing the transistors from typical enhancement mode to depletion mode. Finally, to obtain a better amplifying capability, ZnO TFTs with a *W/L* ratio of 80/5 were selected as the ECoG array for the following experiments. Notably, each transistor array consisted of 16 parallel transistors through a symmetrical layout technique (red frame labeled as shown in Figure 1C and the top‐right inset of Figure S2, Supporting Information) to avert the systematic mismatch caused by the process.^[^ ^35^ ^]^

The electrical properties of the ZnO‐TFT electrode array were measured, as shown in Figure S4C, Supporting Information. The output characteristics of the ZnO‐TFT electrodes with *W/L* = 80/5 exhibited good performance in both the linear and saturation regions (Figure 2B), which is consistent with the simulation results (Figure S3C, Supporting Information). The transfer characteristic curves and corresponding transconductance curves of 12 electrodes in one ZnO‐TFT array are illustrated in Figure 2C and D, respectively, demonstrating good consistency among transistors on the same substrate. Negligible hysteresis was observed in the transfer curve under a cyclic sweep of gate voltage (Figure S5, Supporting Information). According to the simulation, the transconductance attained its maximum value when the gate‐source voltage (*V*_G_) was approximately 4.5 V (Figure S3B, Supporting Information). However, from the measurements, we found that the uniformity of *g*_m_ of ZnO TFTs was relatively poor for *V*_G_ > 3 V. Therefore, as a compromise, in the subsequent experiments, we chose the operating point of *V*_G_ = 2.5 V and drain‐source voltage (*V*_D_) = 5 V, where *g*_m_ was 6.8 µS. Notably, the steady‐state gate current was always less than 0.35 nA for *V*_G_ between −10 and 15 V and *V*_D_ of 5 V (Figure S6, Supporting Information). Additional electrical characteristics can be found in the Supporting Information.

We then conducted mechanical durability tests on the ZnO‐TFT array by bending the device ten times to a curvature of radius of 15 cm and measuring the transfer characteristics of the active electrodes before and after bending (Figure 2E). The transfer characteristic curve was not visibly affected by bending, demonstrating flexibility of the ZnO‐TFT array. The transparency of the ZnO‐TFT electrodes mainly depends on the ITO films. ITO films of the ZnO‐TFT electrodes were annealed at 400 °C in nitrogen after sputtering, which simultaneously increased the transparency and decreased the sheet resistance.^[^ ^34^ ^]^ The average optical transmittance of the device (including the glass substrate) in the visible spectrum (380–780 nm) was higher than 80% (Figure 2F), enabling the device to combine optical stimulation, optical imaging, and electrophysiological recording for the experiments.

### 2.3 In Vitro Evaluation

**Figure 3:**
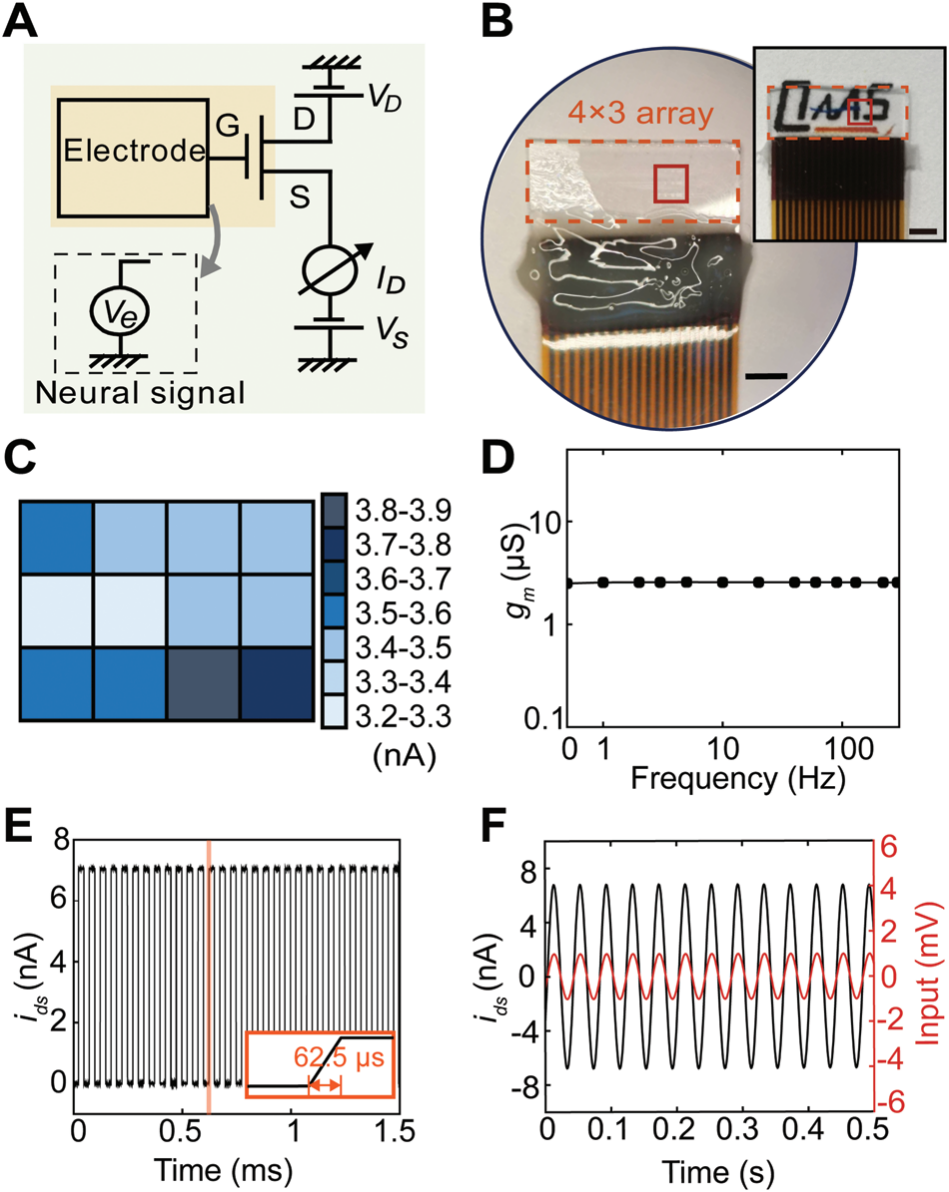
Feasibility of neural signal acquisition of the ZnO‐TFT array. A) Circuit diagram of ZnO‐TFT electrode array. *V*_e_ represents the neural signals from the brain, captured by the gate electrode. The transistors are biased by the voltage sources at the drain (*V*_D_) and source (*V*_S_), and the drain‐source current (*I*_D_) is measured. The colored square labels the brain tissue. B) An image of a 3 × 4 ZnO‐TFT array. Scale bar: 2.5 mm. Inset shows its high transparency. Scale bar: 2 mm. The orange dashed frames of the two images label the edge of the electrode array. The dark red frames label the region of the 12 gate electrodes. C) Distribution of *i*_ds_ from the 12 ZnO TFTs with a sine wave input at a frequency of 60 Hz and 1‐mV *V*_p−p_. D) Frequency response characteristics of the ZnO‐TFT electrode array. E) Response characteristics of a ZnO‐TFT electrode. The response time is less than 62.5 µs. F) Sine wave recorded by the transparent ZnO‐TFT electrode in saline solution with an input of a 25‐Hz frequency and 2‐mV *V*_p−p_ sine wave.

As shown in **Figure** **3**A, neural signals could be collected by the ZnO‐TFT electrodes, whereas the alternating voltage difference (*V*_e_, electrophysiological signals) between the gate and source could control the drain‐to‐source current of the ZnO TFTs (*I*_D_). Then, the *I*_D_ was obtained by a customized recording system, and the alternating component (*i*_ds_) was obtained by a high‐pass filter with the cut‐off frequency at 0.2 Hz.

In the inset of Figure 3B, an optical image of the ZnO‐TFT electrode array distinctly demonstrates its high transparency. When a 60‐Hz, 1‐mV peak‐to‐peak voltage (*V*_p‐p_) sinusoidal wave was transmitted to the 12 electrodes of a 3 × 4 ZnO‐TFT array (Figure 3B), the amplitude of each electrode could be obtained, as shown in Figure 3C. The amplitudes of the ZnO TFTs were mainly in the range of 3.4–3.6 nA, indicating a good uniformity of the ZnO‐TFT electrodes. The ZnO‐TFT electrodes have a flat frequency response characteristic curve (Figure 3D), which means that the ZnO‐TFT electrodes are suitable for mapping neural signals with complex frequency components. Figure S7, Supporting Information, shows a typical recording for an input of a 60‐Hz, 1‐mV *V*_p‐p_ sine wave. Its SNR was calculated to be 16.8 dB (2.50 nA/0.36 nA, RMS). In addition, latency time was measured by sending a rectangular wave to the gate electrodes and recording the output. Figure 3E shows that the delay time of the ZnO‐TFT electrodes was less than 62.5 µs (temporal resolution at a sampling rate of 16 kHz), indicating that the ZnO‐TFT electrodes could record neural signals at a sampling rate of 16 kHz, meeting the requirements of neural electrophysiological recording.

The stability of the electrical performance of ZnO TFTs in liquid or ionic solutions is important for the proposed application. Based on a previous study that summarized the failure time of atomic layer deposited (ALD) ultrathin Al_2_O_3_ films with different thicknesses in water,^[^ ^36^ ^]^ we used an ALD ultrathin Al_2_O_3_ film with a thickness of 20 nm as the encapsulation layer to protect the ZnO transistors. The input sine wave and recorded signal are shown in Figure 3F, when the electrodes were completely soaked in saline. The signal recorded by ZnO‐TFT electrodes was in phase with the input signal, and the SNR was 19.5 dB (RMS noise level of 0.51 nA and RMS amplitude of signal of 4.81 nA). The same electrode was retested one week later and signals with similar SNR were obtained, as shown in Figure S8, Supporting Information. This demonstrates that the ZnO TFT electrodes immersed in the ionic liquid work well with stable electrical properties. The electrical properties of our ZnO‐TFT electrode array, stored at 15 °C and 26% relative humidity for 6 months, were tested (Figure S9, Supporting Information). The results showed that the ZnO‐TFT electrode array maintained a stable electrical performance for an extended time period with no visible deterioration.

### 2.4 In Vivo Electrophysiological Measurements and Optogenetics

**Figure 4:**
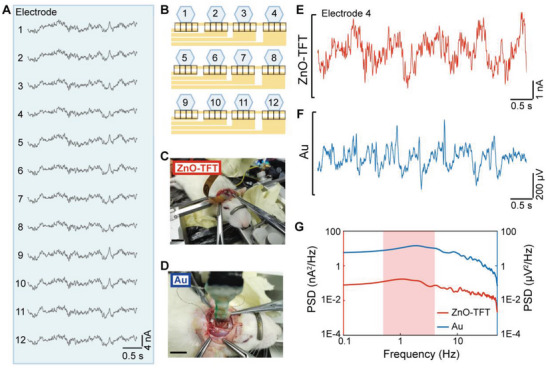
Electrophysiological recording of a ZnO‐TFT array on a rat brain. A) Electrophysiological signals from 12 electrodes of a ZnO‐TFT array. B) Position of 12 electrodes. C) An image of the setup for recording sleep waveform from a transparent ZnO‐TFT array implanted in an anesthetized rat brain. Scale bar: 1 cm. D) An image of the setup for recording sleep waveform from an Au array implanted at the same sites. Scale bar: 1 cm. 5 s long traces of electrophysiological signals recorded by E) one ZnO‐TFT electrode (Electrode 4) and by F) one Au electrode from the anesthetized rat brain. G) Signal power spectral density of neuronal electrical activity in (E, F).

The ZnO‐TFT electrode array was implanted in the brain of an anesthetized rat to record brain activity. As shown in **Figure** **4**, a ZnO‐TFT array and an Au array were placed on the same cortical region of the open skull window for comparative experiments. Figure 4A shows the 12‐channel ECoG signals of the ZnO‐TFT array when the rat was anesthetized, and the distribution of the channels is shown in Figure 4B. Cortical electrical potentials from the two electrode arrays (Figure 4E,F) showed typical episodes of non‐rapid eye movement sleep characterized by high‐amplitude local field potential (0.5–4 Hz) slow waves (i.e., *δ* waves).^[^ ^37^ ^]^ The power spectral density analysis (Figure 4G) of both the ZnO TFTs and Au array electrodes exhibited high power in the low‐frequency oscillations of the *δ* band, which is associated with sleep‐like rhythms. The signals recorded after the death of the rat were considered as noise. Figure S10, Supporting Information, shows the electrophysiological recordings and noise levels obtained using the two arrays. The SNR of the ZnO‐TFT array electrode was 19.9 dB with an RMS amplitude of 1.69 nA for the ECoG signal and 0.17 nA RMS for the noise, whereas the SNR of the Au electrode array was 13.2 dB (70.7 µV RMS for ECoG signal, 15.5 µV RMS for noise). The noise level of 0.17 nA RMS at a band of 0.2–500 Hz corresponds to the input‐referred noise level of 25 µV RMS. The ZnO‐TFT array exhibited a superior SNR compared to that of the Au electrode array. These results suggest that our ZnO‐TFT electrode array could record high quality electrophysiological signals from the cortex of anesthetized rats.

Transparent neural electrode arrays enable penetration of light to directly modulate cortical neurons through the interface with negligible optical power loss, enabling in situ and simultaneous combination of optogenetics with neural electrophysiology. Therefore, for simultaneous optogenetic modulation and neural electrophysiological recording, evaluation of light‐induced artifacts that can contaminate the neural signals is necessary. The Becquerel effect^[^ ^22^ ^]^ is considered as the main cause of photoinduced artifacts. According to previous studies, no difference was found in the transfer characteristics of ZnO TFTs under dark and bright (wavelength range from visible to near ultraviolet) conditions, except for a moderate hysteresis under illumination of 405 nm wavelength.^[^ ^31^ ^]^ ZnO TFT devices are not as sensitive to visible light as other amorphous indium gallium zinc oxide TFTs,^[^ ^35^ ^]^ mostly owing to the polycrystalline nature of the ZnO films deposited by ALD.^[^ ^34^ ^]^

To further study the artifacts of ZnO TFTs, we placed a ZnO‐TFT array electrode on agar to mimic the neural electrophysiological recording from the array on the brain surface (Figure S11, Supporting Information). A 473 nm blue laser was used to illuminate the region of the ZnO‐TFT electrode array with a beam spot size of ≈1 mm. The detailed settings can be found in Section 4. The amplitudes of the artifacts were found to depend on the power and pulse width of stimulation light (Figure S12, Supporting Information). The photoinduced artifacts of the ZnO‐TFT electrode increased sharply on laser irradiation and persisted for tens of milliseconds after the laser was turned off, which is similar to the results of previous studies using metal electrodes.^[^ ^38^ ^]^ Further study will be necessary to fully characterize the overall optical‐electrical properties of ZnO TFTs and the source of potential artifacts.

**Figure 5:**
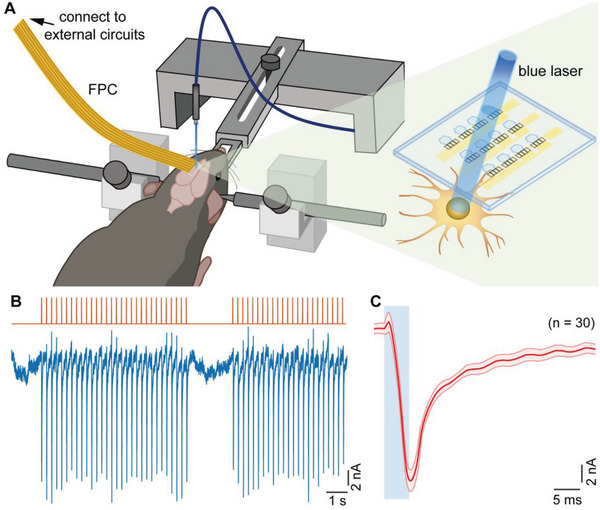
In vivo evaluation of ZnO‐TFT array by laser stimulating on ChR2 virus expressed on mouse brain. A) Schematic of the transparent ZnO‐TFT array on S1 cortical surface (virus injection area) under 473‐nm blue light pulse train stimulation transmitted by an optical fiber. B) Top: the pulse train of optical stimulation (wavelength: 473 nm, frequency: 4 Hz, duration: 5 ms, diameter: 1 mm, and intensity: 13 mW) applied on the injection cortical surface. Bottom: neural signal potential recorded by the transparent ZnO‐TFT electrode array (row 1st, column 4th of the ZnO‐TFT array) exhibiting the light‐evoked negative peaks synchronized with the optical stimulation train. C) Averaged light‐evoked potential (mean ± standard deviation, *n* = 30) of (B). The blue rectangles illustrate the start and duration of optical stimulation pulses.

We then obtained optogenetic and neural recordings using our ZnO‐TFT array. After successful expression of the Channelrhodopsin‐2 (ChR2) virus in the S1 region of the mouse brain, a cranial window around the injection area was drilled and a 3 × 4 ZnO‐TFT electrode array was precisely placed through a stereotaxic instrument to ensure that the electrodes covered the injection site. An image of the implanted ZnO‐TFT array (Figure S13A, Supporting Information) indicates that it could be attached to the mouse cortex, and the clear cortical vessels beneath the array also exhibited the high transparency of the array electrode. The activation of channelrhodopsin with blue optical stimulation depolarizes the neurons.^[^ ^39^ ^]^ As shown in **Figure** **5**A, a 473 nm blue laser light illuminated the electrode region of the ZnO‐TFT array through an optical fiber, similar to the settings in artifact measurement. Because of the high transparency of the ZnO‐TFT array, the laser light could penetrate the array to activate the cortical neurons infected by the ChR2 virus. Figure S13B, Supporting Information, shows an image of the laser beam illuminated on the site on the ZnO‐TFT array laminated on the S1 cortex surface (virus‐injected spot) of the mouse brain. A representative recording from the ZnO‐TFT array in Figure 5B shows the evoked potentials with a larger negative amplitude generated in synchronization with photostimulus pulses (13 mW, 5 ms, and 4 Hz). In contrast to light‐induced artifacts, these potentials have the same frequency as that of the light pulses and similar amplitude with a long persistent tail, demonstrating their reliability as neural signals. The frequency of the light pulses was 4 Hz, which is below the threshold of the ChR2 virus dynamic response. Figure 5C shows the recording of 30 trials (mean ± standard deviation) from Figure 5B with small scattering, indicating the stability of the electrical performance of the ZnO‐TFT array under the same intensity of optical stimulation. The SNR was calculated using the peak value during the period of optical stimulation and baseline without optical stimulation. These values were 19.1 and 0.47 nA, respectively, yielding an SNR of 32.2 dB.

In addition, the effects of the light pulse power and width on the induced neural signals were studied (Figure S14, Supporting Information). As expected, it exhibited an increased neural activation in response to higher photostimulus power and wider pulse width, indicating enhanced depolarization of the neurons. Specifically, a higher optical stimulation power leads to a faster decrease and larger negative peaks of the electrical potential. A wider pulse width results in longer depolarization periods and larger negative peaks of the evoked neural signals.

**Table** **1** summarizes the types of transistors used in various neural electrophysiological interfaces, their optical and electrical characteristics, and the performance of some passive electrodes. Different transistors, such as silicon nanomembrane transistors,^[^ ^40^ ^]^ OECTs,^[^ ^11^ ^]^ and SGFETs,^[^ ^12^ ^]^ have been used in active ECoG arrays. However, because of the opacity of the conductive materials and substrate, these active ECoG arrays are nontransparent. The only active transparent ECoG array was implemented by transparent OECTs, but its transparency is limited to less than 60% owing to the use of metal grids,^[^ ^13^ ^]^ whereas the adoption of ITO in our work ensures a high optical transmittance of over 80% in the visible range. The employment of an Au nano grid realizes a transparency of 73%,^[^ ^41^ ^]^ but it is still lower than the transparency of the ZnO‐TFT array. The higher transparency of ZnO TFTs is more advantageous in optical imaging of the brain, such as single‐ or two‐photon imaging, because the higher transparency makes it possible for fluorescence to pass through the array and be observed. The ZnO‐TFT electrode array exhibited a higher cutoff frequency than that of the transparent OECTs array (50 Hz). Unlike the OECT array, which can measure only a very limited range of low‐frequency signals, the ZnO‐TFT electrode array can measure electrophysiological signals with complex frequency components. Furthermore, the latency time of our ZnO‐TFT array (<62.5 µs) was lesser than that of the transparent OECT array (97 µs). For a transparent active ECoG array, although the transconductance of our ZnO TFTs was approximately three orders of magnitude smaller than that of transparent OECTs based on PEDOT:PSS (2.2 mS maximum),^[^ ^13^ ^]^ the SNR of the ZnO‐TFT electrodes was superior to that of the OECTs, which is attributed to the relatively low gate current of the ZnO TFTs. The SNR of the ZnO‐TFT electrodes was comparable or superior to that of the passive ECoG arrays (Au nano grid, PEDOT:PSS, and Au arrays in Table 1). In addition, unlike OECTs for bioelectronics that face the challenge of electrical performance instability during long‐term storage,^[^ ^42^ ^]^ the long‐term electrical stability of ZnO TFTs during storage has been confirmed,^[^ ^34^ ^]^ demonstrating its superiority, which is particularly useful for the proposed application.

**Table 1:** The comparison of electrical and optical characteristics among our ZnO‐TFT array and various active and passive ECoG arrays

Active/passive	Transistors/materials	Transparency (maximum)	g_m_ (maximum)	SNR for ECoG	Ref.
Active	Silicon nanomembrane transistors	Opaque	∖	34 dB (6.6 mV/45 µV)	[40]
Active	OECTs	Opaque	900 µS	44 dB (1.5 µA/9.5 nA)	[11]
Active	SGFETs	Opaque	3 mS V^−1^	13 dB	[12]
Active	OECTs	60%	2.2 mS	13 dB (0.96 µA/0.2 µA)	[13]
Passive	PEDOT:PSS	Not mentioned	∖	24.2 dB (4.3 mV/0.26 mV)	[11]
Passive	Au nano grid	≈73%	∖	31.7 dB (RMS noise, 90.3 µV)	[41]
Passive	Au	Opaque	∖	13.2 dB (70.7 µV/15.5 µV)	Our work
Active	ZnO TFTs	85%	6.8 µS	19.9 dB (1.69 nA/0.17 nA) 32.2 dB (19.1 nA/0.47 nA)	Our work

### 2.5 7T MRI on a Rat Brain

**Figure 6:**
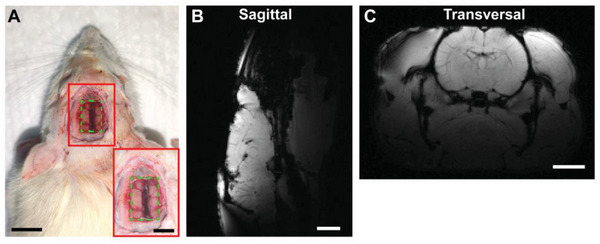
7T MRI study of ZnO‐TFT array on a rat cadaveric model. A) An image of the placement of the ZnO‐TFT array on the surface of rat cadaver brain. Scale bar: 1 cm. Inset: a closer view of the implanted ZnO‐TFT array. Scale bar: 5 mm. The green frame labels the location of the electrode array. B) Sagittal T1‐weighted MRI image of a rat brain. Scale bar: 5 mm. C) Transversal T1‐weighted MRI image of rat brain. Scale bar: 5 mm.

Many practical applications of simultaneous measurements of MRI‐ECoG have been introduced for the study of epilepsy, sleep, and other brain functions.^[^ ^43^, ^44^ ^]^ Implanted ECoGs may introduce artifacts that obscure the regions of diagnostic importance, thus hindering MRI. MRI was conducted with the ZnO‐TFT array on a rat cadaveric model under an ultra‐high magnetic field of 7T. **Figure** **6**A shows an implanted ZnO‐TFT array. The array was tightly attached to the surface of the rat cadaver brain, spanning the left and right hemispheres. Figures 6B,C exhibit the MRI at two slice positions, the sagittal and transverse planes, respectively. Additional MRI images are shown in Figures S16 and S17, Supporting Information. The MRI images showed no blur around the rat cadaver brain tissue, even in the regions close to the array, signifying no effects of the ZnO‐TFT array on MRI. Therefore, the ZnO‐TFT array could be used for simultaneous MRI‐ECoG recording to study the mechanisms of brain network activity, demonstrating its great potential for the application.

## 3 Conclusion

An active transparent electrode array for electrocorticogram electrical recording was proposed and fabricated using ZnO TFTs as the local neural signal amplifier. The employment of ITO and ZnO achieved the highest transparency of active ECoG arrays to date (up to 85%) and enabled in situ and synchronous neural recording and optical stimulation for optogenetics in the mouse brain.

A ZnOTFT fabrication technology compatible with that of the electrode array was developed. To enhance the amplification capability of the ZnO TFTs, the parameters of the ZnO TFTs were optimized by both SPICE model simulation and fabrication. The electrical, optical, and mechanical properties of the ZnO‐TFT electrode arrays were characterized. To further verify the adaptability of our electrode array in recording electrocorticography signals, a series of characterizations was performed to prove its constant level of gain at varied frequencies, extremely low latency times, and consistency in electrical properties. The ability of long‐term storage of up to six months and the waterproof performance of a 20‐nm‐thick Al_2_O_3_ encapsulation were also verified.

The ZnO‐TFT electrode array was implanted in a rat brain for obtaining sleep waveform and on a mouse brain for obtaining optogenetics. The former verified the feasibility of electrocorticography signal recording and the latter proved its high transparency, allowing for optical stimulation in optogenetics. The SNR was enhanced by adopting a preamplifier. For spontaneous electrophysiological activity, the SNR reached 19.9 dB, which is superior to that of passive electrodes (Au). Owing to the large peak of the evoked potentials, a higher SNR of 32.2 dB was gained. Moreover, the array did not blur MRI images at a 7T ultra‐high magnetic field, demonstrating its great potential of MRI‐ECoG synchronous recording. In addition, its high transparency is compatible with optical brain imaging techniques, which can be applied to more studies combining optics and electrophysiology, in the future. As a multimodal active ECoG array, it is a promising tool for monitoring electrophysiological signals with synchronized optical modulation, 7T MRI, and optical imaging.

## 4 Experimental Section

### ZnO‐TFT Circuit Design

The amplifier and address gate circuits were designed based on an n‐type LTPS‐TFT SPICE model (level 62) and simulated using HPICE software (Synopsys). The threshold voltage was set to 0 V according to the authors’ previous study.^[^ ^31^ ^]^

### ECoG Array Fabrication

The processing steps of the ZnO‐TFT electrode array are shown in Figure S1, Supporting Information. The layout of the ZnO‐TFT electrode array was drawn using the Tanner EDA software, as shown in Figure S2, Supporting Information. The ZnO‐TFT circuits and electrode array were integrated on the same substrate using the multi‐layer process discussed in the Supporting Information. Thin‐film ITO was employed in the ZnO TFTs because it has been commonly used in transparent electrode arrays owing to its high transmittance in the visible spectrum, high electrical conductivity,^[^ ^45^ ^]^ and biocompatibility.^[^ ^46^ ^]^ An ALD Al_2_O_3_ film was used as the encapsulation layer, because Al_2_O_3_ is considered to be one of the most biocompatible materials,^[^ ^47^ ^]^ whereas the ALD Al_2_O_3_ film is a pinhole‐free film, which is an excellent electrical insulator and moisture barrier.^[^ ^48^, ^49^, ^50^ ^]^ Finally, the ZnO‐TFT array was bonded with a flexible printed circuit, as described in the Supporting Information.

### Characterization

Electrical properties of the devices were characterized using a semiconductor characterization system (Keysight B1500A) equipped with a probe station. The transfer characteristics of the ZnO TFTs were assessed by applying a *V*_D_ of 5 V and *V*_G_ between −10 and 15 V, and the transconductance was calculated by the differential method. The output characteristic curve was obtained for *V*_D_ between 0 and 10 V and *V*_G_ between −1 and 5 V with a step of 1 V. Mechanical flexibility was assessed under a curvature of radius of 15 cm, the transfer characteristics were measured after 0th and 10th cycles, and the transparency of the ZnO‐TFT array was evaluated using a UV–vis spectrophotometer (Cary 60 Series, Agilent Technologies, Inc.) in the wavelength range of 200–800 nm.

### In Vitro Experiments

Characterization of the frequency response and response time were also conducted on a probe station. For the frequency response tests, a probe was placed on a gate electrode pad to deliver sinusoid waves with a *V*_p‐p_ of 1 mV at a frequency ranging from 0.1 to 300 Hz generated by a weak electrophysiological signal simulator (SKX‐8000 Series, Ming Sheng Electronic Technology Co., Ltd, Xuzhou, China). To characterize the response time, a square wave of 40‐ms duration, 0.5‐mV amplitude, and 50% duty cycle was generated by the simulator and applied to the gate electrode, and the corresponding output signals were recorded at a sampling rate of 16 kHz. For electrical characterization in saline, a dupont line transferred the sinusoid wave (25 Hz and 2‐mV *V*_p‐p_) generated by the signal simulator to a saline solution (0.9% wt) in a beaker. Meanwhile, a ZnO‐TFT electrode array was submerged in the saline for the gate electrode to collect signals transmitted through the saline solution. The output signal from the ZnO TFT was transferred by an *I*–*V* conversion circuit and then recorded by a customized electrophysiological signal recording system.

### Animals

Male Sprague‐Dawley rats (250–300 g) and male C57BL mice (25 g) were used. All surgical and experimental procedures conformed to the Guide for the Care and Use of Laboratory Animals (China Ministry of Health), and were approved by the Animal Care Committee of Zhejiang University, China. Animals were fully acclimatized to the environment for seven days before any experimental procedure and were given standard rat chow and water.

### Electrophysiological Recording on Rat Brain Surface

The rats were first treated with atropine by intra‐abdominal with a dosage of 0.12 mg/100 g to relieve pain and were then anesthetized with propofol (10 mg mL^−1^ solution and 1.2 mL/100 g) via intraperitoneal injection (IP) according to their weight and set in a stereotaxic head frame. An incision was made on the skin of the brain, and a dry and clean skull was exposed after H_2_O_2_ treatment. A cranial nail was screwed onto the posterior fontanelle skull as the ground. A cranial window was drilled to expose a 7 × 5 mm^2^ window in the right hemisphere. Mannitol was injected by IP to prevent edema. The ZnO‐TFT electrode array was fixed on the arm of the dorso‐ventral axis, and the implanted position was carefully adjusted using a micro‐drive screw with a 3D axis. Neural signals were recorded using the customized recording system described above. After data acquisition, the ZnO‐TFT electrode array was removed and an Au electrode array was implanted in the same position for neural signal recording. The rats were then euthanized. After the deaths of the rats, the ZnO‐TFT electrode array and Au electrodes were implanted in the same region, and the noise was recorded by the two arrays separately.

### Optogenetics

AVV‐hSyn‐hChR2(H134R)‐mCherry virus was injected in primary somatosensory cortex (S1, AP: −1.2 mm, ML: +2.2 mm) of a male mouse. Two weeks were required by the mouse to recover from the surgery and for viral expression. For optogenetics, the mouse was anesthetized and placed in a stereotaxic head frame. A cranial window was created around the virus‐injected site using an electrical cranial drill, and the dura was removed. ZnO‐TFT electrode array was implanted into the cranial window, with the gate electrode positioned precisely in the virus‐injected region. The ground wire was connected to a cranial nail that was fixed to the posterior fontanelle. A blue laser of 473 nm wave length (Shanghai Laser & Optics Century Co., Ltd., China) was used to irradiate the gate electrode region. Each light pulse train was triggered by an electrical stimulator (Master‐8, A.M.P.I Instruments, Ltd., Israel), which had 30 trials at 4 Hz, with a pulse width varying from 3 to 20 ms and an excitation power of 1, 5, 13, and 40 mW.

### Light‐Induced Artifacts

A ZnO‐TFT electrode array was placed on agarose, and the reference/ground wire was inserted into the agarose. Laser stimulation with different pulse widths and powers was applied to the gate electrode region, and the evoked potential was measured using a recording system.

### MRI

A piece of ZnO‐TFT array was placed on the surface of the rat cadaver brain across the left and right hemispheres. The dura of the rat brain was removed and saline was applied to keep the brain hydrated. MRI was recorded using a 7T research scanner (Magnetom, Siemens Healthcare, Erlangen, Germany). The rat brain was placed in the head coil of a 7T MRI scanner. The sequence had a TR of 410 ms, TE of 7.56 ms, and a slice thickness of 1 mm.

### Data Acquisition and Processing

All recordings were obtained in the unipolar mode at a sampling frequency of 1 kHz unless specified and filtered with a high‐pass filter of 0.2 Hz (four orders, Butterworth) and a notch filter of 50 Hz in MATLAB (MathWorks). For optogenetics and light‐induced artifacts, the evoked signals from 30 trials were averaged for assessment.

## Conflict of Interest

The authors declare no conflict of interest.

## Supporting information

Supporting Information

## Data Availability

The data that support the findings of this study are available from the corresponding author upon reasonable request.
