# Supplementary material for: Multimodal Electrocorticogram Active Electrode Array Based on Zinc Oxide‐Thin Film Transistors
Source: Adv Sci (Weinh). 2022 Nov 20;10(2):2204467. doi: 10.1002/advs.202204467 (PMC9839861; doi:10.1002/advs.202204467)
Supplement: Supplementary file 1 — Supporting Information [file ADVS-10-2204467-s001.pdf]

Supporting Information

**Multimodal electrocorticogram active electrode array based on zinc oxide-thin film transistors**

*Fan Zhang, Luxi Zhang, Jie Xia, Wanpeng Zhao, Shurong Dong*, Zhi Ye*, Gang Pan, Jikui Luo, Shaomin Zhang**

F. Zhang, S. Zhang
Key Laboratory of Biomedical Engineering of Ministry of Education
Qiushi Academy for Advanced Studies
Zhejiang Provincial Key Laboratory of Cardio-Cerebral Vascular Detection Technology and Medicinal Effectiveness Appraisal
Zhejiang University
38 Zheda Road, Hangzhou, 310027, China
Email Address: shaomin@zju.edu.cn

L. Zhang, S. Dong
College of Information Science and Electronic Engineering
Frontier Center of Brain Science and Brain-machine Integration, Cancer Center
Zhejiang University
38 Zheda Road, Hangzhou, 310027, China
Email Address: dongshurong@zju.edu.cn

J. Xia, W. Zhao, Z. Ye, J. Luo
College of Information Science and Electronic Engineering
Zhejiang University
38 Zheda Road, Hangzhou, 310027, China
Email Address: yezhi@zju.edu.cn

G. Pan
College of Computer Science and Technology
Zhejiang University
38 Zheda Road, Hangzhou, 310027, China

## Experimental Section

*Multi-layer process:*

A 4-inch, highly transparent Corning Eagle XG Glass (500 μm thick) was used as the substrate. The first ITO layer ($In_2O_3$: $SnO_2$ = 90wt%:10wt%) of 100 nm thickness was sputtered and patterned by photolithography as the source and drain of the transistors. ITO was then treated by rapid thermal annealing (RTA) at 400 °C for 5 min in nitrogen environment to achieve high transparency and electrical conductivity.[1, 2] A ZnO active layer of 20 nm thickness was deposited by thermal atomic layer deposition (ALD, Lesker 150LX) at 200 °C and then annealed at 400 °C for 5 min in oxygen ambient. An $Al_2O_3$ layer of 10 nm thickness was deposited by ALD to protect the ZnO layer and then annealed in oxygen at 200 °C for 30 s. Then, the ZnO layer was patterned by photolithography and wet etching to form the conductive channel of the TFTs and annealed at 400 °C for 5 min in oxygen ambient. The second $Al_2O_3$ layer of 20 nm thickness, as the gate dielectric layer, was deposited by ALD and annealed at 200 °C for 30 s in oxygen environment. Through-holes were patterned and punched by wet etching on the second $Al_2O_3$ layer, and then the sample was annealed at 400 °C for 5 min in nitrogen environment. Then, the gate electrodes and bonding pads of a 100-nm ITO layer were formed by photolithography and lift-off process via sputtering and annealed at 400 °C for 5 min in oxygen ambient. Deuterium ions were injected into the transistors via plasma-enhanced chemical vapor deposition (70 W, 15 min) to adjust the threshold voltage of the ZnO TFTs.[3, 4] Finally, a third $Al_2O_3$ layer of 20 nm thickness was deposited on the entire device except the gate electrodes and bonding pad region, which was used as the protective layer of the whole device to prevent the entry of moisture and oxygen in air from penetrating into the device, which deteriorated the device properties.

## Electrical characteristics

*Mobility:*

The overdrive voltage ($V_{GT}$) is defined from the gate-source voltage ($V_G$) and threshold voltage ($V_T$) as:

$$V_{GT} = V_G - V_T \quad \text{(S1)}$$

If $V_{GT} > 0$, the drain current ($I_D$) in the linear region and saturated region can be expressed as:

$$I_D = \mu_{FET} C_{ox} \frac{W}{L} \frac{V_{GT}^2}{2}, V_D \geq V_{GT} \quad \text{(S2)}$$

Where $\mu_{FET}$ denotes the mobility, $C_{ox}$ is the gate oxide capacitance per unit area, $W/L$ is the aspect ratio of TFT and $V_D$ is the drain-source voltage.

The effective mobility of the TFTs is calculated according to Equation S2. For the fabricated ZnO TFTs, $C_{ox}$ = 160 nF cm$^{-2}$ and $W/L$ = 80/5. The threshold voltage of the TFTs is -0.7 V (see Figure 2C, E). When $V_G$ = 1 V and $V_D$ = 10 V, the drain current is 29 μA. Therefore, the effective mobility of the TFTs at the saturation region is 7.8 cm$^2$ V$^{-1}$s$^{-1}$.

*Resistance :*

The resistance between drain and source ($R_t$) of ZnO-TFT electrodes is 11.3 kΩ, the resistance of source ($R_S$) and drain ($R_D$): $R_S = R_D = 0.5R_t$ = 5.65 kΩ.

*Parasitic capacitance:*

The capacitances of gate ($C_g$) of each ZnO TFT with $W/L$ = 5/5 is 160~180 fF. For our ZnO-TFT electrode array, the $W/L$ of TFTs is 80/5, so the capacitance becomes 16 times that of $W/L$ = 5/5, i.e. $Cg = 160 fF \times 16 = 2.56 pF$ .

The parasitic capacitance which is the result of interaction between the gate electrode and drain ($C_{gd}$), and the gate electrode and source ($C_{gs}$). This can be calculated as:

$$Cgd = Cgs = \frac{1}{2} Cg = 1.28 pF \qquad \text{(S3)}$$

**Supporting Figures**

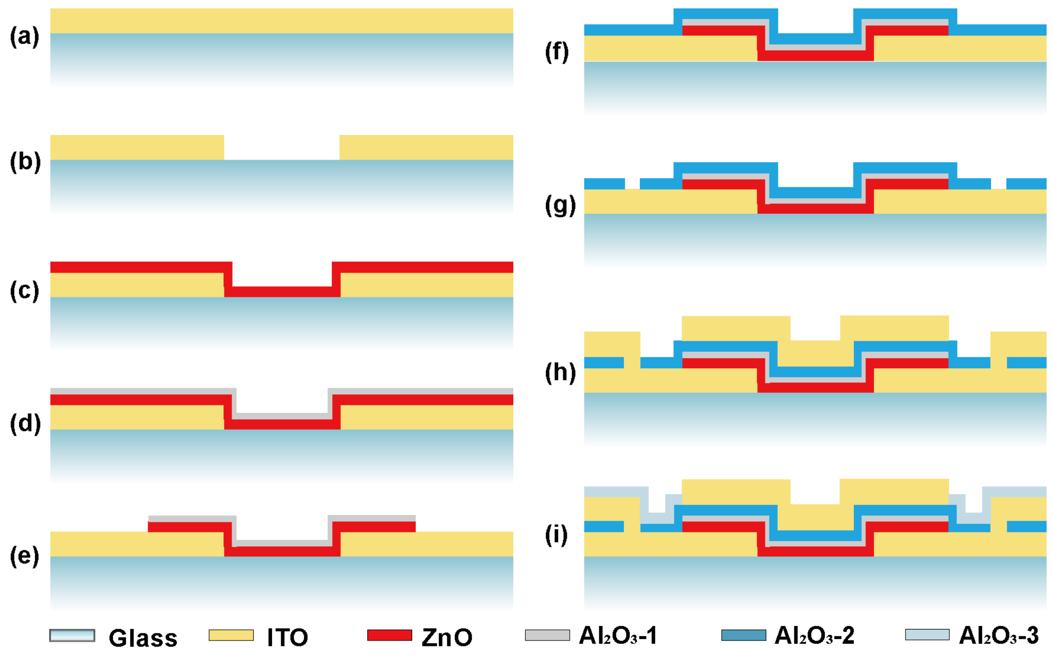


**Figure S1.** The fabrication process of ZnO-TFT electrode array.

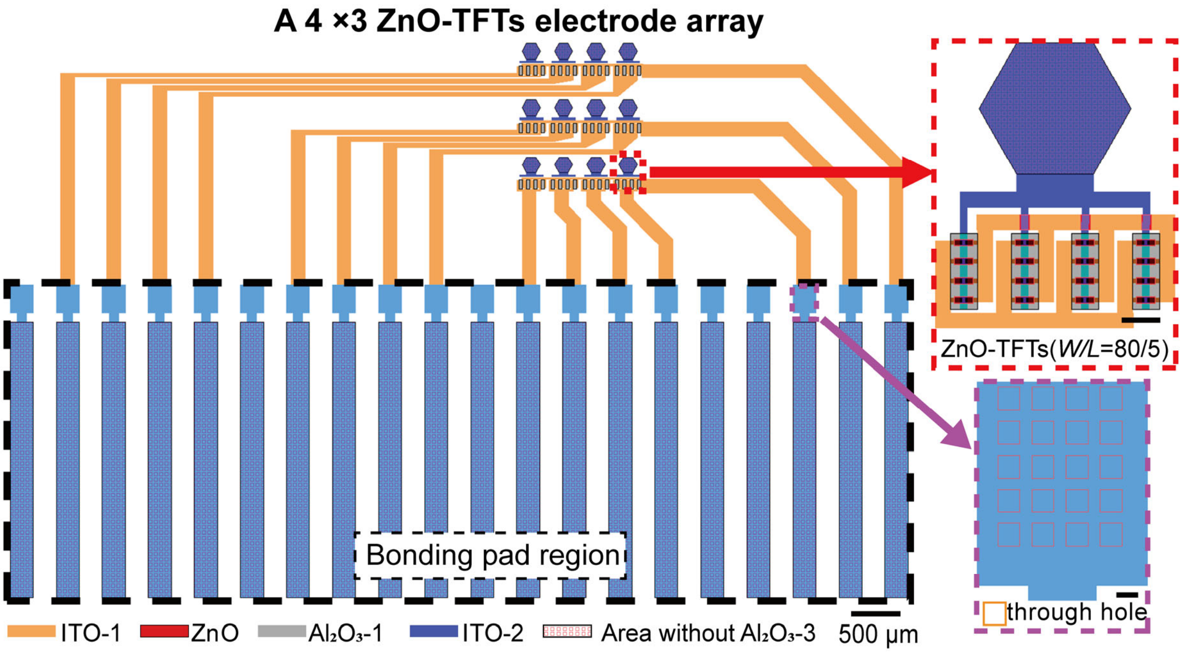


**Figure S2.** The layout of a 3 × 4 ZnO-TFT electrode array. The second $Al_2O_3$ layer covered the whole device except for the through holes. The inset diagram at the top right is an

electrode with a ZnO-TFT of *W/L* = 80/5. Scale bar: 50 μm. The inset diagram at the bottom right shows the through holes connected source/drain and bond pads. Scale bar: 10 μm.

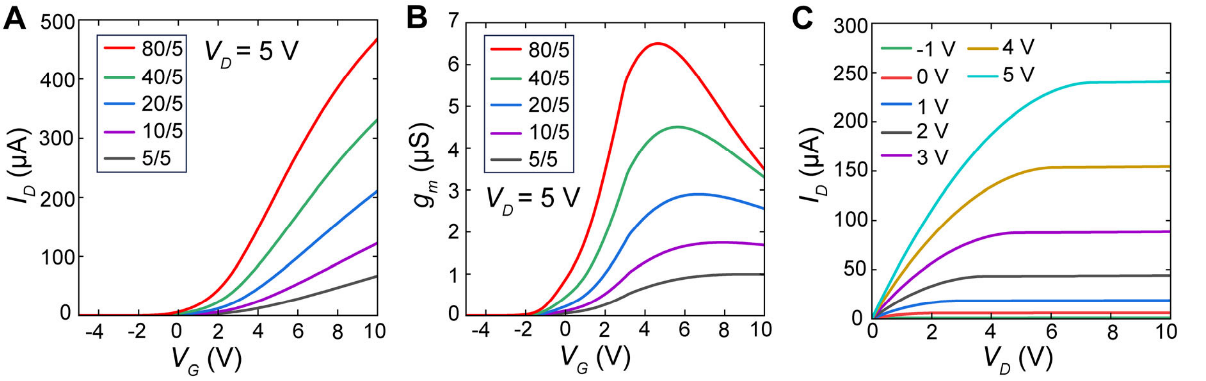


**Figure S3.** Electrical performance simulation for ZnO TFT through n-type LTPS-TFT SPICE model (level 62). (A) The transfer characteristic curves are simulated by ZnO TFT with various width to length ratios (*W/L*). (B) The transconductance curves calculated from the transfer characteristics of panel A. (C) The output characteristic curves simulated by ZnO TFT with *W/L*=80/5, with drain voltage ($V_D$) and gate voltage ($V_G$) varying from -1 V to 5 V (step = 1 V).

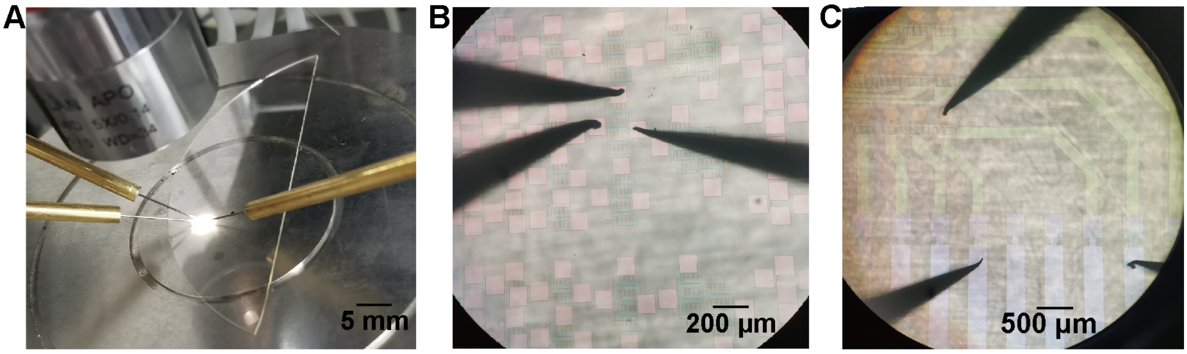


**Figure S4.** (A) The photograph of ZnO TFT measurement on the probe station system. (B) Microscopic image of ZnO TFT (*W/L*=80/5) measurement on the probe station system. (C) Microscopic image of 3 × 4 ZnO-TFT electrode array measurement on the probe station system.

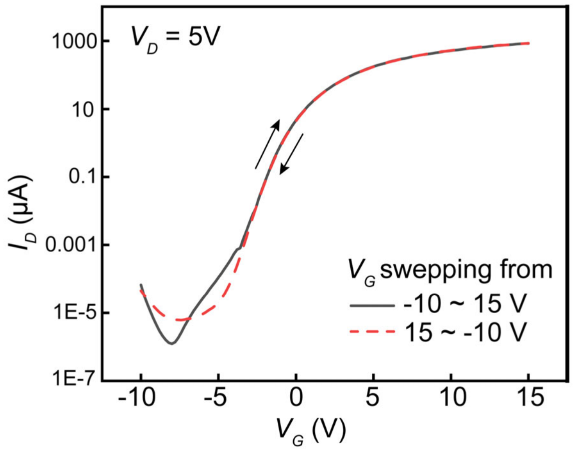


**Figure S5.** Transfer characteristics under cyclic scanning of the gate voltage showing negligible hysteresis of ZnO-TFT electrodes.

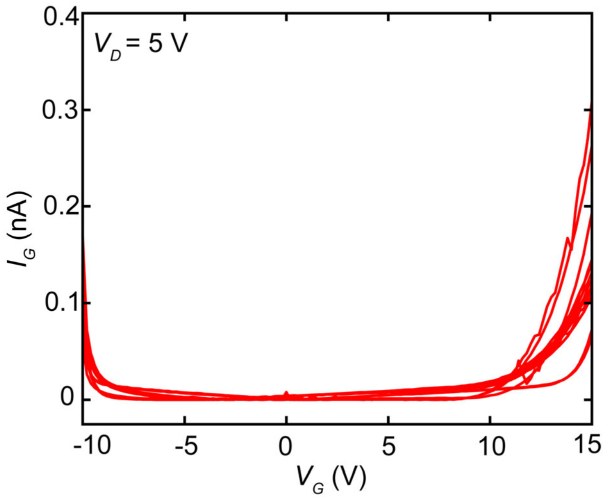


**Figure S6.** The steady state gate current (n=12) measured with $V_G$ between -10 V and 15 V, and $V_D$ of 5 V.

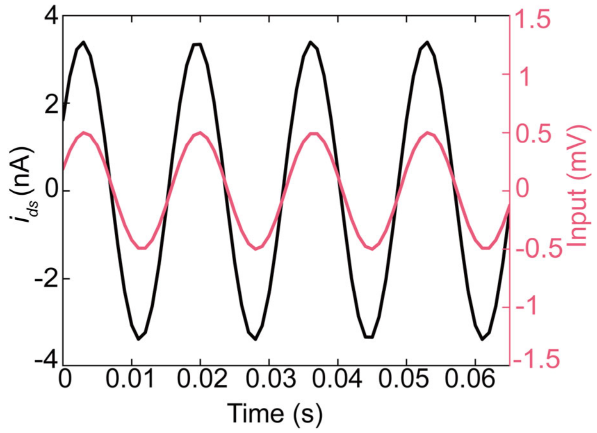


**Figure S7.** The sine wave with a frequency of 60 Hz was acquired by the ZnO-TFT array. Input: 60 Hz, peak-to-peak voltage ($V_{p-p}$) of 1 mV, sine wave.

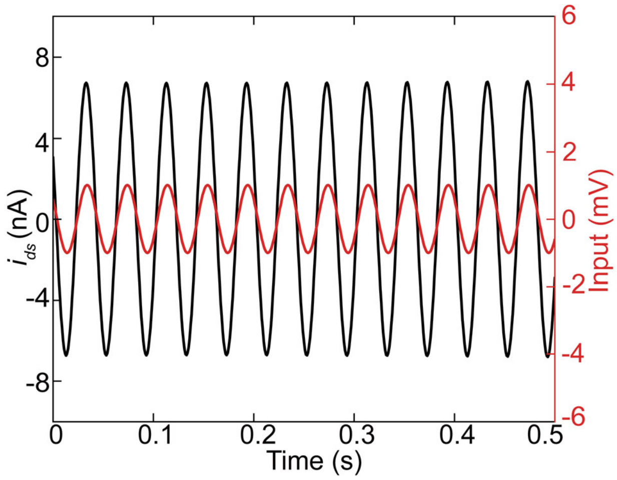


**Figure S8.** The 25 Hz sine wave is acquired by the ZnO-TFT electrode array that has been soaked in saline solution one week ago. Input: 25 Hz, $V_{p-p}$ of 2 mV, sine wave.

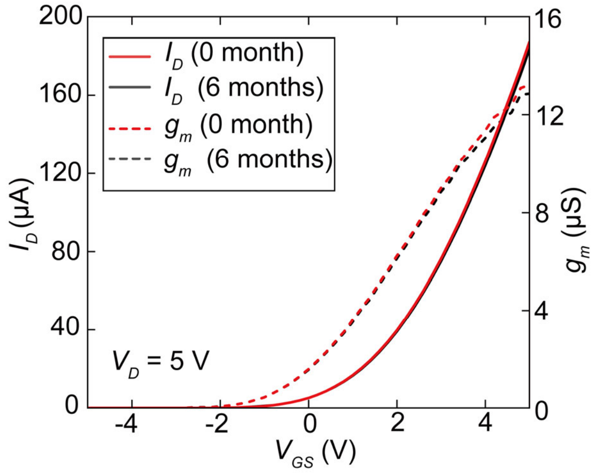


**Figure S9.** Electrical properties of a ZnO-TFT electrode stored in dry environment for 6 months, demonstrating the electrical stability of ZnO-TFT electrodes.

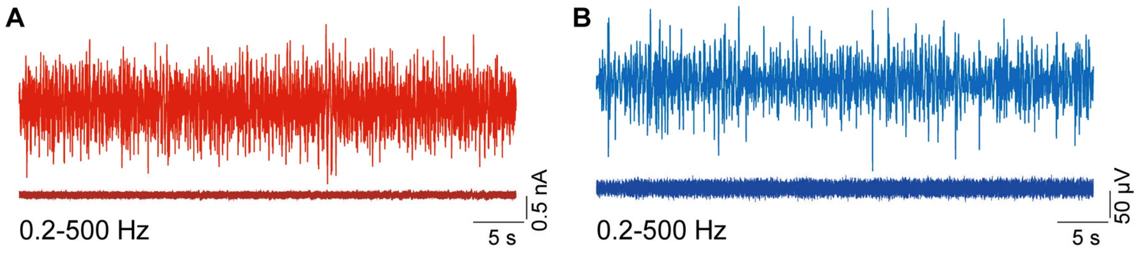


**Figure S10.** (A) Electrophysiological signal from one ZnO-TFT electrode (top) and noise recorded on died rat brain (bottom). (B) Electrophysiological recording from one Au electrode (top) and noise recorded on died rat brain (bottom). The bandwidth of ECoG signals and noise is 0.2-500 Hz.

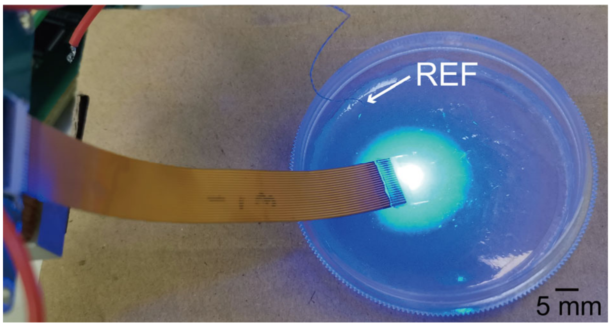


**Figure S11.** Light-induced artifacts evaluation by positing ZnO-TFT electrode array on agar and applying continuous light pulse stimulation. The reference wire is labeled with a white arrow.

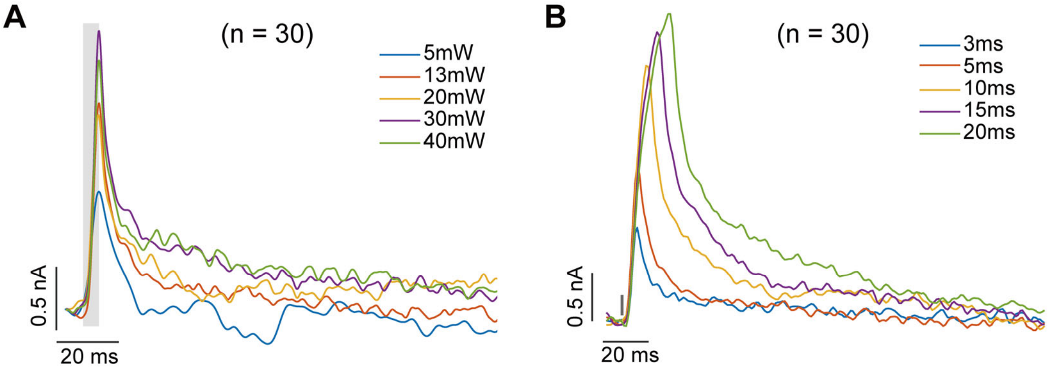


**Figure S12.** Averaged light-induced artifacts of ZnO-TFT electrode array. (A) Averaged light-induced artifacts (n = 30) under light stimulation with various power (4 Hz, 5 ms). The gray rectangles illustrated the start and duration of light stimulation pulses. (B) Averaged light-induced artifacts (n = 30) under light stimulation with different pulse width (4 Hz, 13 mW). The gray line labled the start of light stimulation pulse.

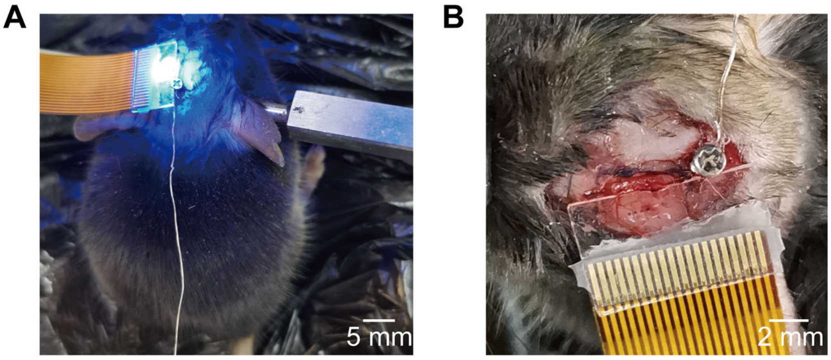


**Figure S13.** (A) The image of an optogenetic experiment using ZnO-TFT electrode array. (B) The ZnO-TFT electrode array was posited on the brain surface of a mouse.

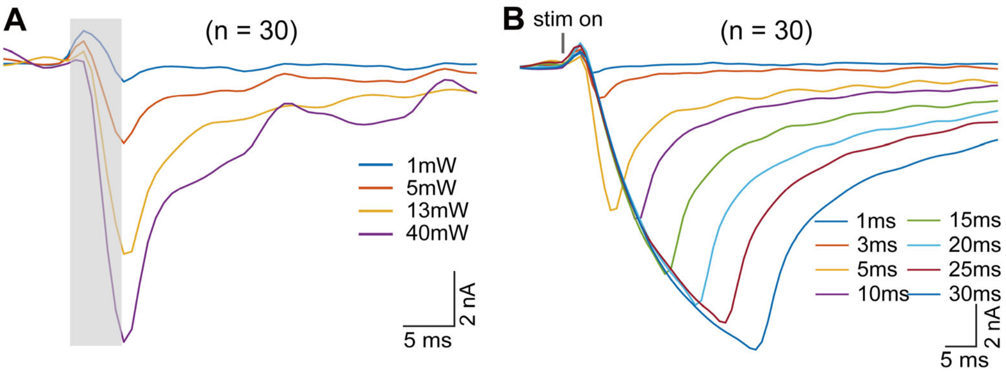


**Figure S14.** Averaged light-evoked potential (n = 30) recorded by ZnO-TFT electrode under photostimulus with different light power (A) and duration (B). In (A), 5 ms, and 4 Hz light pulse trains were applied, and the gray rectangles illustrated the start and duration of light stimulation pulses. In (B), 13 mW, 4 Hz light pulse trains were applied. The gray line labeled the start of the light stimulation pulse.

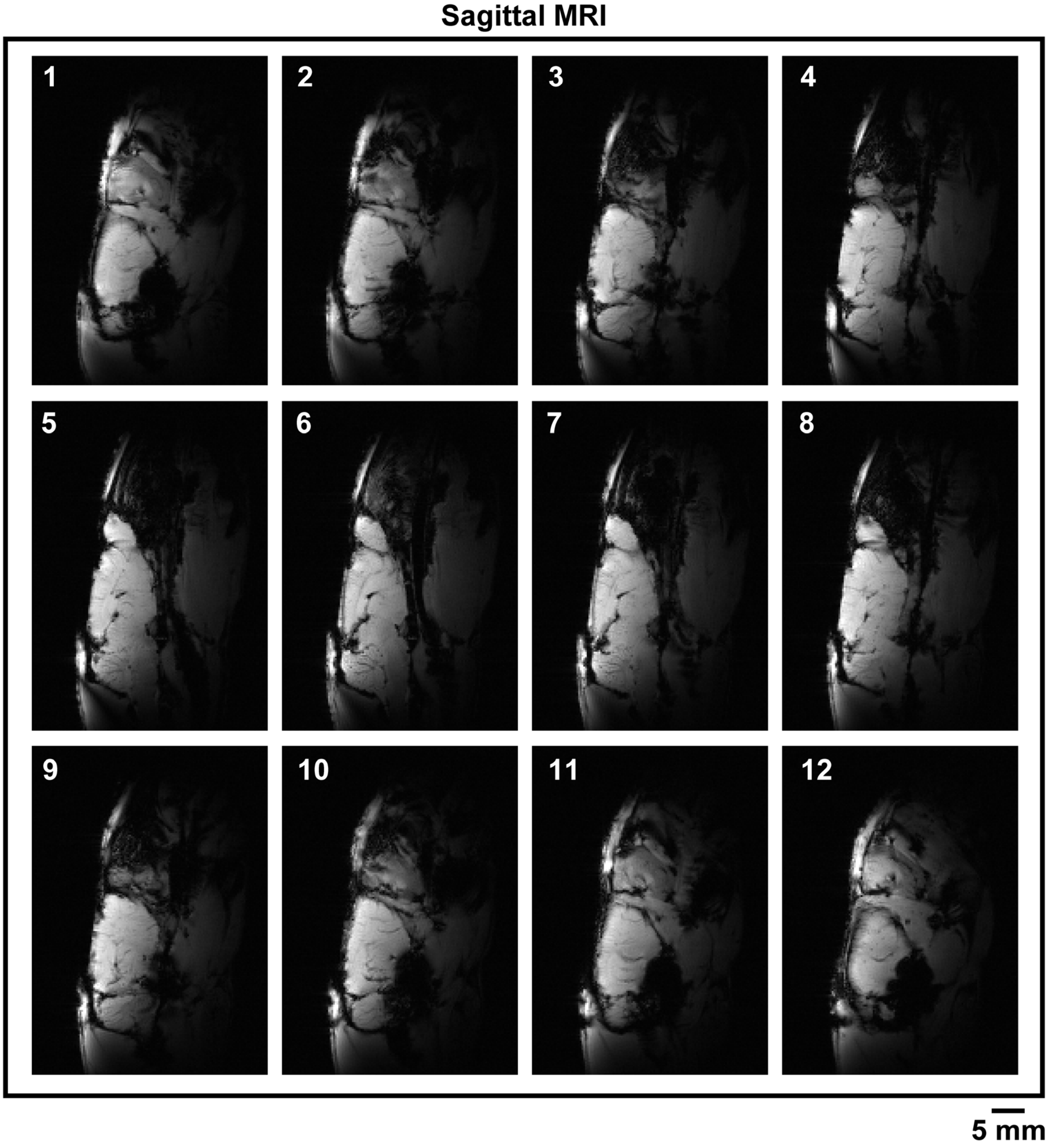


**Figure S15.** Sagittal T1-weighted MRI image of a rat brain. Slice thickness: 1 mm.

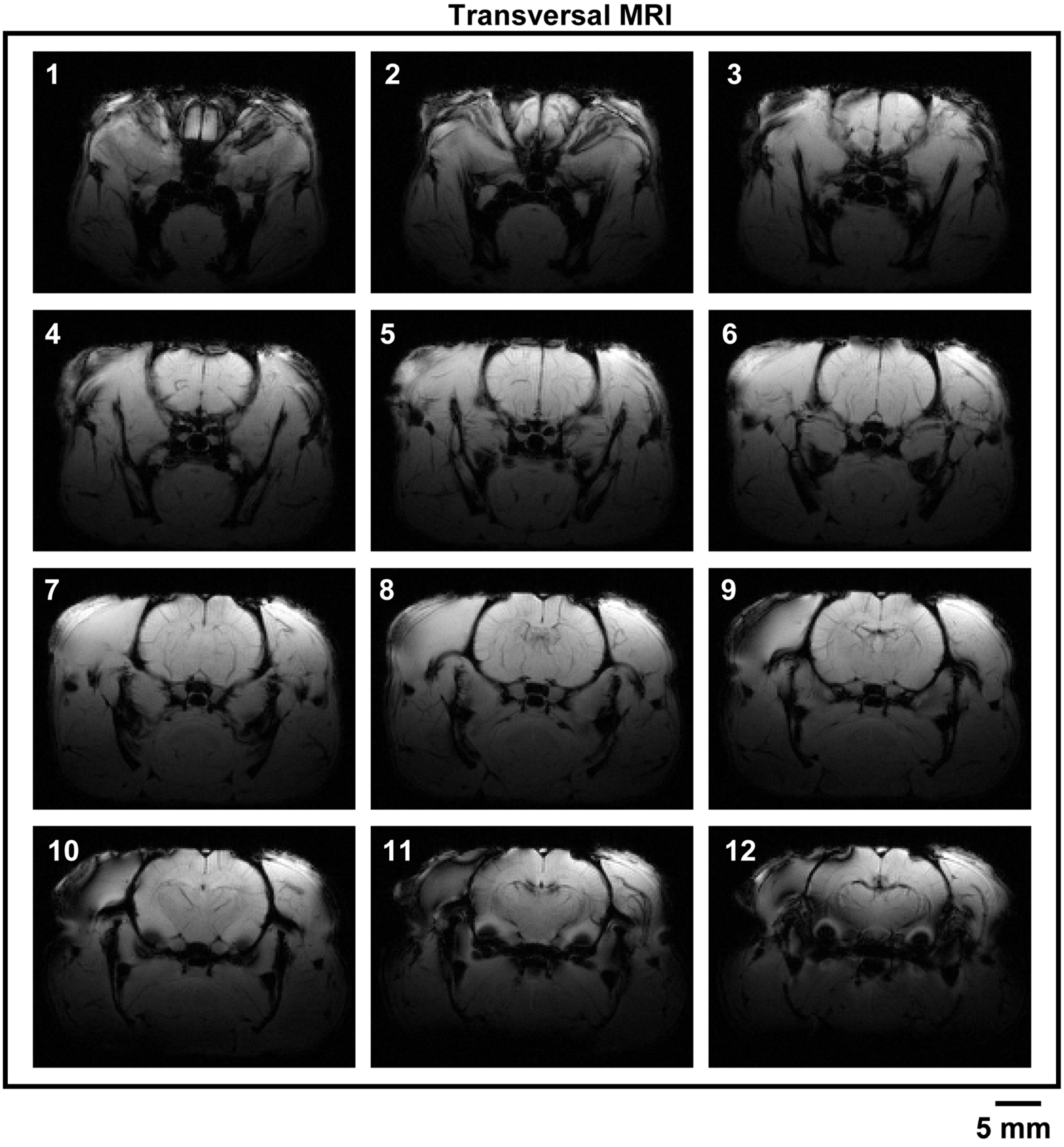


**Figure S16.** Transversal T1-weighted MRI image of a rat brain. Slice thickness: 1 mm.

**References**

[1] Z. Ye, H. Xu, T. Liu, N. Liu, Y. Wang, N. Zhang, Y. Liu, *IEEE Trans. Electron Devices* **2017**, *64*, 4114.

[2] M. Gao, R. Job, X. De-Sheng, W. Fahrner, *Chin. Phys. Lett.* **2008**, *25*, 1380.

[3] H. Xu, Z. Ye, N. Liu, Y. Wang, N. Zhang, Y. Liu, *IEEE Electron Device Lett.* **2017**, *38*, 1383.

[4] W. Zhao, L. Han, N. Zhang, X. Zhang, S. Dong, Y. Liu, Z. Ye, *IEEE Electron Device Lett.* **2020**, *41*, 1508.
